# Data on bond strength of resin cement systems to CAD/CAM resin composite after aging

**DOI:** 10.1016/j.dib.2021.107474

**Published:** 2021-10-12

**Authors:** Masaki Yagi, Toshinori Okawa, Fumiaki Kawano

**Affiliations:** a Yagi Dental Clinic, Shiga 520-2132, Japan; b Department of Comprehensive Dentistry, Graduate School of Biomedical Sciences, Tokushima University, Tokushima 770-8504, Japan

**Keywords:** CAD/CAM resin composite, Bond strength, Resin cement, Primer, Thermocycle

## Abstract

CAD/CAM resin composite crowns are inexpensive tooth-colored prostheses that have been widely used. However, bonding between CAD/CAM resin composites and resin cements could be difficult since the resin composite is highly cross-linked. There is limited existing data on the resin cements’ bond strength with different monomers to CAD/CAM resin composites. In this study, CAD/CAM resin composite was bonded to an SUS rod with three different resin cements following treatment of the bonding surface using the manufacturer's recommended primer. After storing the specimens in water at 37 °C for 24 h, half of them were tested immediately and half were thermocycled for 10,000 cycles in water for a dwell time of 20 s at 5 and 55 °C. The means of the tensile bond strength and standard deviations were determined for each resin cement and testing condition. The data were compared using two-way ANOVA and Bonferroni's multiple comparison tests at 95% confidence level.

## Specifications Table

**Table:** 

Subject	Biomaterials
Specific subject area	Dental Materials
Type of data	Table Figure
How data were acquired	Tensile bond strength was measured using a universal testing machine. Data was analyzed using a statistical software, EZR (Saitama Medical Center of Jichi Medical University).
Data format	Raw Analyzed
Parameters for data collection	Combination of three types of commercial resin cements (G-CEM ONE EM, Multi Bond II, and Super-Bond) and three types of a manufacturer's recommended primers (Ceramic Primer, Universal Primer, and Super-Bond PZ Primer, respectively).
Description of data collection	The manufacturer's recommended primer was applied to the CAD/CAM resin composite. They were bonded using three different types resin cements. Half of all the specimens were tested immediately following 24 h of immersion in water at 37°C (thermaocycling 0 cycle; TC 0), and half were subjected to thermocycling 10,000 cycles following 24 h of immersion in water. The tensile test was used to determine the bond strength in MPa. The fracture surface and mode between resin cement and CAD/CAM resin composite were determined by naked eye and scanning electron microscope observation.
Data source location	Department of Comprehensive Dentistry, Graduate School of Biomedical Sciences, Tokushima University, 3-18-15 Kuramoto-cho, Tokushima-city, Tokushima 770-8504, Japan
Data accessibility	Mendeley Data: https://data.mendeley.com/datasets/jd8y573s4r/3

**Value of the Data**

• The data would contribute to the expansion of clinical adaptation for the CAD/CAM crown in the dental field.
• The data, bond strength between the dental CAD/CAM resin composite and resin cements is important for long-term success in prosthodontic treatments since it prevents debonding failure.
• The data could help dentists in the selection of an appropriate resin cement system when the CAD/CAM resin composite is bonded to the abutment tooth in prosthodontic treatments.
• The data could be compared with other collected data for various types of dental material, including composites and hard resin artificial teeth.

## 1 Data Description

**Fig. 1:**
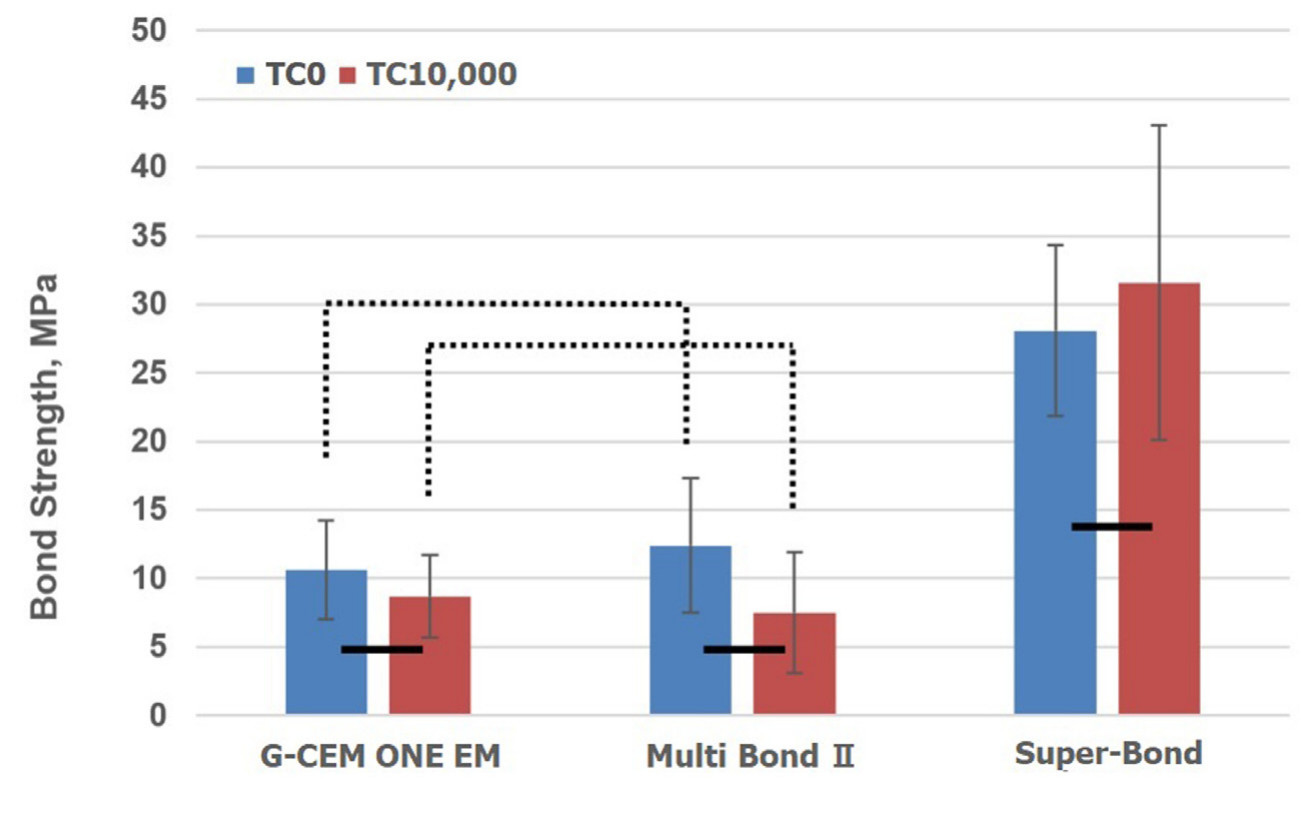
Tensile bond strength of CAD/CAM resin composite to the SUS rod. Bars connected by line were not significantly difference between thermocycles (*p* > 0.05) Bars connected by the dotted lines were not significantly different among the resin cements (*p* > 0.05).

**Fig. 2:**
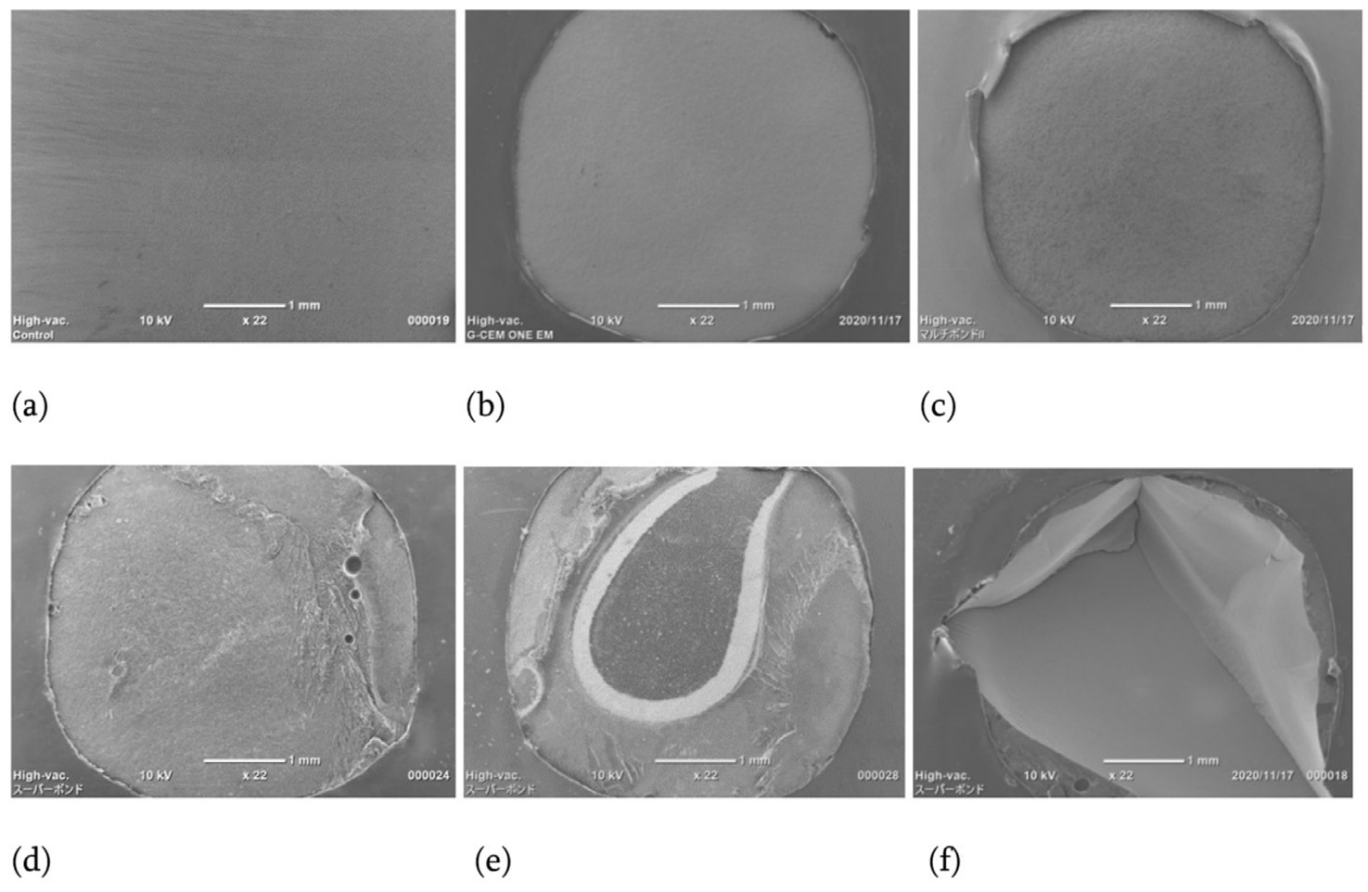
Representative scanning electron microscope (SEM) image of fracture mode following tensile bond strength test (Original magnification X 22). (a) Control: surface before tensile bond strength test, (b) G-CEM ONE EN: adhesive failure at CAD/CAM resin composite/cement interface, (c) Multi Bond II: adhesive failure at CAD/CAM resin composite/cement interface, (d) Super-Bond: cohesive failure in resin cement, (e) Super-Bond: mixture of cohesive failure at CAD/CAM resin composite/cement interface, (f) Super-Bond: cohesive failure in CAD/CAM resin composite.

**Fig. 3:**
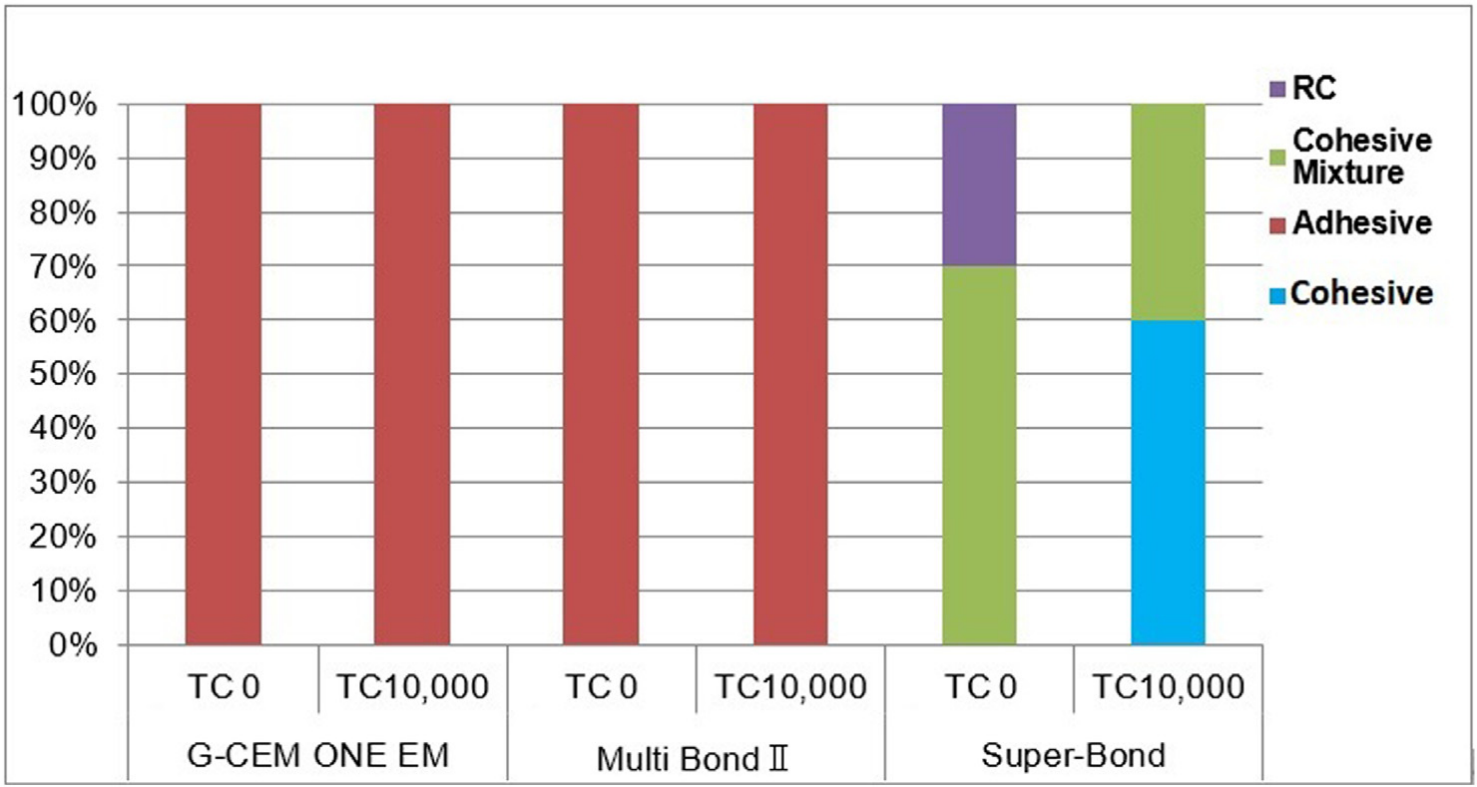
Fracture mode following tensile bond strength test of CAD/CAM resin composite at 0 and 10,000 thermocycles.

The datasets provide information on the bonding properties (tensile bond strength and fracture mode) of the CAD/CAM resin composite to SUS 304 steel (Lot. No 181013, Yamamoto Seiki LLC, Shiga, Japan) using commercially available resin cements and manufacturer's recommended primers. The resin cement, manufacturer's recommended primers, and CAD/CAM resin composite used in this test are listed in Tables 1, 2, and 3, respectively. These primers included Ceramic Primer II for G-CEM ONE EM, Universal Primer for Multi Bond II, and Super-Bond PZ Primer for Super-Bond. Fig. 1 shows the bond strength between the CAD/CAM composite and the SUS rod using three different resin cements before and after thermocycling (TC). The bond strengths were compared using two-way analysis of variance and Bonferroni's multiple comparison test at a 95% confidence level. The results are shown in Fig. 1. The fracture mode of the resin cement to the CAD/CAM resin composite is illustrated in Figs. 2 and 3.

**Table 1:** Commercial available resin cements used in this test.

Brand Name	Manufacturer	Constituent	Lot No.	Composition	Ref.
G-CEM ONE EM	GC Corporation, Tokyo Japan	Translucent	1911121	Paste A: fluoro alumino-silicate glass, methacrylic acid ester, initiator	[1]
				Paste B: silica filler, methacrylic acid ester, phosphate ester monomer, initiator	
Multi Bond II	Tokuyama Dental Inc., Tokyo, Japan	Powder	028C1	PMMA	[2]
		Liquid	046	MMA, UDMA, HEMA, borate initiator, MTU-6	
		Primer	045B	acetone, water, phosphate ester monomer, UDMA, initiator	
Super-Bond	Sun Medical Co Ltd., Shiga, Japan	Polymer powder bulk-mix teeth color	LF1T	PMMA	[3]
		Quick monomer liquid	TK1	4-META, MMA	
		Catalyst V	SV32	TBB	

PMMA: poly (methyl methacrylate), UDMA: urethane dimethacrylate, HEMA: hydroxy ethyl methacrylate.

MTU-6: 6-methacryloyloxyhexyl 2-thiouracil-5-carboxylate, 4-META: 4-methacryloxyethyl trimellitate anhydride,

MMA: methyl methacrylate, TBB: Tri-n-butyl borane.

**Table 2:** The primers used in this test.

Trade Name	Manufacturer	Lot number	Composition	Ref.
Ceramic Primer II	GC Corporation, Tokyo Japan	1905081	vinyl silane, methacrylic acid ester, ethanol	[4]
Universal Primer	Tokuyama Dental Inc, Tokyo, Japan	Liquid A: 017C	ethanol, MTU-6, γ-MTPS	[5]
		Liquid B:016E	aceton, phosphate ester monomer, MAC-10, UDMA	
Super-Bond PZ Primer	Sun Medical Co LTD, Shiga, Japan	Liquid A:ST12	MDP, MMA	[3]
		Liquid B:SG12	γ-MPTS, MMA	

MAC-10:11-methacryloxy-1,1-undecane dicarbonic acid, MDP: 10-methacryloyloxydecyl dihydrogen phosphate, MMA: methyl methacrylate, γ-MPTS: 3-methacryloxypropyl trimethoxysilane

**Table 3:** CAD/CAM resin composite.

Brand Name	Manufacturer	Lot No.	Composition	Ref.
Cerasmart 300 A2	GC Corporation, Tokyo Japan	1801221	UDMA, barium glass	[6]

UDMA: urethane dimethacrylate

The raw data of the tensile bond strength and fracture mode is uploaded on MendeleyData.

## 2 Experimental Design, Materials and Methods

### 2.1 Sample preparation

A total of 60 CAD/CAM resin composite blocks with a thickness of 4 mm and a diameter of 20 mm were prepared and polished with SiC #600 polishing paper; 50 µm air abrasive treatment (0.2 MPa for 5 sec per specimen) was applied uniformly to the bonding area of the CAD/CAM resin composite and the SUS rod, and washed with tap water for 10 min and air dried.

After attaching a masking tape with a hole of a thickness of 50 µm and a diameter of 4.8 mm on the polishing surface of the CAD/CAM resin composite, the primer was applied to the area of the hole for 5 s and air dried. The SUS rods were bonded using three different resin cements to the CAD/CAM resin composite and maintained at room temperature for 30 min after setting the SUS rod on the CAD/CAM resin composite. After all the specimens were stored in water at 37 °C for 24 h, half of them were tested immediately (no thermocycled; TC 0) and half were thermocycled for 10,000 cycles (TC 10,000) in water for a dwell time of 20 s at 5 and 55 °C.

The resultant specimens were used for the tensile bond strength test. (*n* = 10)

### 2.2 Tensile bond strength test

The tensile bond strength between the CAD/CAM resin composite and resin cement was measured using a universal testing machine (AGS-H, Shimadzu Corp., Kyoto, Japan) at a crosshead speed of 2 mm/min.

The maximum load was recorded when the SUS rod was removed from the CAD/CAM resin composite surface. The bond strength was calculated on dividing the maximum load by the bonding area.

### 2.3 Determination of fracture mode

The fractured surface of the CAD/CAM resin composite specimens following the tensile bond strength test was observed by naked eye; scanning electron microscope (SEM) was used to determine the fracture mode. Representative SEM images of the fracture mode following the tensile bond strength test is shown in Fig. 2.

Fig. 2a: Surface of the CAD/CAM resin composite specimen prior to the tensile bond strength test, Fig. 2b and c: Adhesive failure at the CAD/CAM resin composite-resin cement interface, Fig. 2d: Cohesive failure in the resin cement, Fig. 2e: Mixture of cohesive failure at the resin cement-CAD/CAM resin composite interface, and Fig. 2f: Cohesive failure in the CAD/CAM resin composite. The ratios of the fracture modes are shown in Fig. 3.

### 2.4 Statistical analysis

The bond strengths were analyzed using the statistical software EZR (Saitama Medical Center, Jichi Medical University, Saitama, Japan). The means of the tensile bond strength and standard deviations were determined for each resin cement and testing conditions (*n* = 10). The data were compared using two-way ANOVA and Bonferroni's multiple comparison tests at a 95% confidence level.

## CRediT authorship contribution statement

**Masaki Yagi:** Writing – original draft, Writing – review & editing, Formal analysis. **Toshinori Okawa:** Writing – original draft, Writing – review & editing, Formal analysis. **Fumiaki Kawano:** Supervision, Writing – review & editing.

## Declaration of Competing Interest

The authors declare that they have no known competing financial interests or personal relationships that have or could be perceived to have influenced the work reported in this article.
